# Opening the policy blackbox: unravelling the process for changing national diagnostic and treatment guidelines for vivax malaria in seven countries

**DOI:** 10.1186/s12936-021-03959-w

**Published:** 2021-10-30

**Authors:** Varunika Ruwanpura, Josselyn Neukom, Koen Peeters Grietens, Ric N. Price, Kamala Thriemer, Caroline A. Lynch

**Affiliations:** 1 Global Health Division, Menzies School of Health Research and Charles Darwin University, PO Box 41096, Casuarina, Darwin, NT 0811 Australia; 2 Independent Consultant, Ho Chi Minh City, Vietnam; 3 Institute of Tropical Medicine, Antwerp, Belgium; 4 School of Tropical Medicine and Global Health, Nagasaki University, Nagasaki, Japan; 5 Mahidol-Oxford Tropical Medicine Research Unit (MORU), Faculty of Tropical Medicine, Mahidol University, Bangkok, Thailand; 6 Centre for Tropical Medicine and Global Health, Nuffield Department of Clinical Medicine, University of Oxford, Oxford, UK; 7 Medicines for Malaria Venture (MMV), Geneva, Switzerland

**Keywords:** *Plasmodium vivax* malaria, Malaria health policy, Policy pathways, Radical cure, Malaria elimination

## Abstract

**Background:**

The changing global health landscape has highlighted the need for more proactive, efficient and transparent health policy-making. After more than 60 years of limited development, novel tools for vivax malaria are finally available, but need to be integrated into national policies. This paper maps the malaria policy-making processes in seven endemic countries, to identify areas where it can be improved to align with best practices and optimal efficiency.

**Methods:**

Data were collected during a workshop, convened by the Asia Pacific Malaria Elimination Network’s Vivax Working Group in 2019, and subsequent interviews with key stakeholders from Cambodia, Ethiopia, Indonesia, Pakistan, Papua New Guinea (PNG), Sri Lanka and Vietnam. Documentation of policy processes provided by respondents was reviewed. Data analysis was guided by an analytic framework focused on three a priori defined domains: “context,” “actors” and “processes”.

**Results:**

The context of policy-making varied with available funding for malaria, population size, socio-economic status, and governance systems. There was limited documentation of the process itself or terms of reference for involved actors. In all countries, the NMP plays a critical role in initiating and informing policy change, but the involvement of other actors varied considerably. Available evidence was described as a key influencer of policy change; however, the importance of local evidence and the World Health Organization’s endorsement of new treatments and diagnostics varied. The policy process itself and its complexity varied but was mostly semi-siloed from other disease specific policy processes in the wider Ministry of Health. Time taken to change and introduce a new policy guideline previously varied from 3 months to 3 years.

**Conclusions:**

In the medium to long term, a better alignment of anti-malarial policy-making processes with the overall health policy-making would strengthen health governance. In the immediate term, shortening the timelines for policy change will be pivotal to meet proposed malaria elimination milestones.

## Background

The global health community has to contend with the evolution of old and new diseases, rapid Research & Diagnostic (R&D) pipelines and a renewed drive for transparency and accountability endorsed within the Sustainable Development Goals [1]. Although these dynamic challenges highlight an urgent need for proactive, forward looking, and innovative policy processes [2–4], policy-making in general is a diffuse, opaque and difficult to define process [5–8]. Specific to malaria, several studies have identified that the process of changing national treatment guidelines is complex and often unclear [9–11]. As new and potentially highly impactful tools near the end of the R&D pipeline and become available, facilitating the uptake into policy and practice becomes a key consideration [12]. A prime example is the management of vivax malaria, in which a range of new tools and approaches are finally ready for the market after more than six decades of limited development [13–15].

Vivax malaria accounts for approximately 7.5 million to 15 million clinical cases annually, with most cases occurring in the Asia Pacific region followed by the Americas and the Horn of Africa [16–19]. The relative proportion of *Plasmodium vivax* is increasing, since unlike *Plasmodium falciparum*, *P. vivax* forms dormant liver stages (hypnozoites) that can reactivate (relapse) weeks to months after initial infection. More than 65% of recurrent *P. vivax* malaria is caused by reactivation of these dormant liver forms [20]. Thus prevention of *P. vivax* relapses has potential to contribute significantly to global and regional elimination efforts, especially in endemic countries which have set ambitious targets to eliminate the parasite by 2030 [21].

The World Health Organization (WHO) and most vivax-endemic countries recommend treating both the blood and liver stage of the parasite [22, 23]—referred to as “radical cure”. Currently, the only widely available hypnozoitocidal drug is primaquine (PQ). PQ and other 8-aminoquionolines can induce haemolysis in patients with glucose-6-phosphate dehydrogenase (G6PD) deficiency [24]. The absence of adequate point of care (PoC) tests to identify patients at risk of haemolysis has certainly limited the roll out of radical cure and led to low prescription rates [25, 26]. Furthermore since G6PD testing is not routinely available in most endemic countries, nearly all national malarial control programmes recommend a low dose treatment regimen over 14 days (3.5 mg/kg total dose) to minimize the risk of drug induced haemolysis, and yet this has significantly lowered anti-relapse efficacy in some regions [27]. The limited use of PQ is exacerbated by the lack of a pre-qualified paediatric PQ formulation [28] and low adherence to the full 14 day course resulting in reduced effectiveness [29, 30].

A range of new diagnostic tools and treatment options are now available to overcome these logistical constraints. Short course high dose PQ [31] and single dose tafenoquine (TQ) have potential to overcome adherence issues [32, 33], and when combined with a novel point of care G6PD diagnostic can be prescribed safely. However, experiences from previous health policy change processes suggest that the time lag between the availability of evidence and policy development is 7 to 10 years [34], and this excludes subsequent delays in implementation. To shorten this timeline, it is imperative that a better understanding is gained of the steps in the policy change processes, the factors that influence those steps and the elements along the policy change pathway. Surprisingly few studies have investigated malaria policy processes, and those that have been conducted have focused on sub-Saharan Africa [9, 10, 35–41], with only one study in Latin America [42] and another in Asia [43].

This paper, therefore, maps the pathways of malaria policy processes in seven vivax endemic countries, taking a prospective approach to identify important areas for improvement to ensure best practice and timely policy-making.

## Methods

### Analytical framework

The analytical framework was developed based on work by Walt and Gilson [44], which has been widely applied in health policy analysis and Tesfazghi [35], which adapted the framework to malaria-related policy-making. It focuses on three domains for data collection and analysis: context, actors, and processes. At the analysis stage, an additional domain was added to accommodate the emerging theme of “towards power and evidence” in the cross-country comparison (Table 1). The context domain encompasses funding, level of available documentation for policy-making, and the overall socio-economic status of countries. Actors were considered individuals or organizations pivotal to the policy processes. Identifying the people involved in policy change, and the nature of their relationships is key to understanding non-technical factors that influence change. Finally, since most policy-making processes are considered opaque and complex, greater clarity as to the steps that should be involved should enable identification of where novel policy processes or innovations could be implemented.

**Table 1:** Analytical framework adapted from Walt 1994 and Tesfazghi 2016 [35, 44]

Context	Country context regarding socio-economic status & current malaria treatment context and availability of documents outlining actors and process as defined below
Actors	Stakeholders/individuals that make or influence malaria treatment policy
Process	The way policies are developed and approved, and the respective timelines
Towards power and evidence	Power is characterized by authority, finances and access to knowledge. Evidence is defined as *‘Any form of knowledge, including, but not confined to research, of sufficient quality to be used to inform decisions’ Buse *et al*. 2012* [78]

### Data collection

Data were collected in three phases: (i) during a workshop conducted in 2019 by the Asia–Pacific Malaria Elimination Network's (APMEN) Vivax Working Group (VxWG) (ii) through email exchanges and subsequent interviews with National Malaria Programme (NMP) representatives and additional stakeholders including WHO country partners as well as local and international research partners in 2020 and (iii) through a review of select documentation provided by NMPs and other stakeholders interviewed as well as collation of contextual data to highlight the different country contexts in regards to malaria elimination and socio-economic context.

#### *Workshop*

The role of the APMEN VxWG has been described previously [45]. In October 2019, the annual meeting was held in Kathmandu, Nepal, following which a one-day workshop was held with NMP representatives. Participants were divided into nine discussion groups (with 2–3 country representatives per group). Each group was allocated a non-NMP facilitator and note taker. Workshop sessions covered several topics, key among which was group work to outline national policy pathways to change treatment guidelines for malaria. For this, participants developed flowcharts identifying steps in the national policy process and key stakeholders involved in decision-making for each of their countries. Participants were asked to consider whether pathways might differ for the introduction of new drugs, for example, TQ compared to shortening an already existing PQ treatment regimen that is currently recommended by the WHO for use over 14 days.

#### *Interviews*

Workshop outputs were used to develop follow-up questions for a more in-depth multi-country analysis of malaria policy processes. The latter were incorporated into semi-structured interviews with NMP representatives and, where possible, other key stakeholders including WHO country officers and global health partners including research partners. Interviews focused on eliciting information on the timelines and specific steps in each country’s national policy change process, and composition and influence of key stakeholders at each stage of the policy change process.

NMP interviewees were identified by purposive sampling based on attendance at the workshop. For non-NMP respondents, a mix of snowball sampling based on recommendations from the NMP interviews and purposeful sampling based on professional connections of the research team through ongoing or previous public health research in countries was used.

In early March 2020, an initial email was sent to all NMP representatives who attended the workshop along with country-specific follow-up questions and a request for an additional interview. If no reply was received within approximately 1 week, two additional reminders were sent. NMP representatives who answered the request were then invited for zoom or telephone interviews. Where possible, interviews with non-NMP stakeholders were conducted. If needed, follow-up interviews with the same respondents were scheduled after the initial data analysis. All interviews were conducted by telephone or zoom meeting between 26 March and 9 May 2020. A summary of each country’s policy pathway was shared with NMP representative interviewees for review and further input prior to inclusion in the multi-country analysis. The final country policy pathways have, therefore, been further developed and crosschecked for accuracy by interviewed NMP respondents.

#### *Document review*

All respondents were asked to provide documentation of the national policy pathway and relevant policy decision-making bodies for example, terms of reference (ToR) for Technical Working Groups and other committees responsible for informing or approving policy change. A limited number of documents were obtained from Cambodia, Indonesia and Sri Lanka [46–48]. These consisted of the ToR of Cambodia’s Malaria Diagnosis and Treatment technical working group, the Indonesian Ministry of Health’s (MoH) decree to its Diagnosis and Treatment of Malaria Working Group (translated from Bahasa) and the Sri Lankan National Strategic Plan for Prevention of Reintroduction of Malaria in Sri Lanka 2018–2022. Further contextual data collation was done to elucidate country contexts regarding the status of treatment guidelines, elimination target and the overall socio-economic context in which national malaria policy-making takes place. This was done through a brief review of literature for each country.

### Data analysis

During the 2019 workshop, participants generated conceptual flowcharts mapping pathways for policy change and identifying relevant stakeholders to the policy change process in their individual countries. Participants were asked to consider different pathways for TQ and PQ policy revision. Post-meeting these flowcharts were analysed by four authors (JN, VR, CAL, KT) to identify gaps or unclear elements and were cross checked with additional notes taken during the session by the facilitator and/or notetaker.

These preliminary malaria policy maps were used to generate a more in-depth interview (IDI) guide for follow-on interviews with NMP representatives and other stakeholders. Any unclear aspects identified in the initial flowcharts were queried in the IDIs. Interview notes were manually coded in line with the analytical framework. Themes identified from interview transcripts as relevant to the dimensions of actors and process were used to further develop policy pathway maps. Using an iterative process, summary results and policy maps were sent for review to respondents to clarify remaining questions and verification. Where available, sourced documents were used to provide additional context and triangulation of findings obtained through the workshop and interviews.

## Results

### Participants

Over 40 NMP members and other country representatives from 20 countries attended the 2019 APMEN VxWG workshop. At least one attendee per country was contacted in March 2020 and invited to participate in follow-up interviews. A total of seven (32%) NMPs responded to interview invitations within the allotted timeframe and met virtually with the research team. These were representatives from seven countries: Cambodia, Ethiopia, Indonesia, Pakistan, Papua New Guinea (PNG), Sri Lanka and Vietnam. Interviewees included senior representatives from national malaria programmes (8 from Cambodia, Ethiopia, Indonesia, Pakistan and PNG), WHO country officers (1), national public health institute representatives and international malaria researchers with in-depth country experience (3).

## Country specific findings

### Cambodia

#### *Context*

The socio-economic context and the current anti-malarial treatment policies are summarized in Table 2. Overall, the funding for malaria control activities has declined from 2011 to 2014 and then increased modestly by 2017. The majority of funding for malaria activities comes from external resources with Global Fund and USAID/PMI being the largest contributors [18]. There was no available guidance for the policy change process in Cambodia from the National Malaria Programme (Cambodia’s National Center for malaria, CNM) or by the Ministry of Health. There is a documented Terms of Reference (ToR) for NMP’s Diagnosis and Treatment Working Group (DTWG)—a body significantly involved in Cambodia’s malaria policy-making process. The DTWG ToR outlines this group’s responsibility for revising policy based on scientific evidence and clearance from the NMP [46].

**Table 2:** Country context

Country	Population size, 2019 [75]	GDP—per capita (PPP) [79] (2017 est.) in US$	Population at risk of malaria (%), 2019 [75]	Elimination target	Proportion of *P. falciparum*, *P. vivax* [18]	Funding dominance external vs domestic [18]	Treatment Guidelines	Last update of guidelines	Comment
Cambodia	16,486,542	$4000	11,659,118 (71%)	*P. falciparum* by 2023 and all species of malaria by 2025 [80, 81]	*P. falciparum*: 58% *P. vivax*: 41%	External	PQ14 (low dose) without G6PD testing	2014 guidelines were reviewed, but not changed, in 2017	While the current guidelines do not require G6PD testing (despite the WHO report stating), qualitative G6PD testing is being used in a pilot involving 88 public health facilities in 4 provinces between November 2019 and September 2020. This pilot is expected to inform the 2020 review of the guidelines
Ethiopia	112,078,736	$2200	76,213,540 (77%)	2030 [82]	*P. falciparum*: 69% *P. vivax*: 30%	External	PQ14 (low dose) without G6PD testing	2018	
Indonesia	270,625,584	$12,400	270,625,584 (100%)	2030 [81]	*P. falciparum*: 63% *P. vivax*: 37%	Approx. 40% domestic	PQ14 (low dose) without G6PD testing		
Pakistan	216,565,320	$540	212,907,532 (98%)	2030 [81]	*P. falciparum*: 21% *P. vivax*: 78%	Nearly 50% domestic	PQ14 (low dose) G6PD testing recommended where possible, but not required (although 2018 WHO report states it as a requirement)	2018	Guidelines will next be updated after the 2020 therapeutic efficacy study results are available
Papua New Guinea	8,776,119	$370	8,776,119 (100%)	2030 [81]	*P. falciparum*: 76% *P. vivax*: 23%	External	PQ14 (low dose) without G6PD testing	2011	Research is planned in 2020–2021 to inform the next version of the guidelines which—pending research findings and WHO recommendations—may include PQ7 and G6PD testing for confirmed vivax cases
Sri Lanka	22,889,201	$1290	0 (0%)	Elimination reached in 2016 [83]	NA		PQ14 (low dose) with qualitative G6PD testing	2016	Next planned review of the guidelines will take place in 2021, depending on the 2020 mid-term review findings and timeline
Vietnam	96,462,116	$6900	71,091,518 (74%)	*P. falciparum* by 2020 and all species of malaria by 2030 [81]	*P. falciparum*: 64% *P. vivax*: 35%	Approx. 75% external	PQ14 (low dose) without G6PD testing	2016	The 2018 review resulted in a decision that no updates were required. New guidelines are currently pending review with approval from the MoH expected in 2020 and will recommend but not require G6PD testing where possible, before treatment with PQ14

#### *Actors: responsibility for initiating, reviewing and approving policy change*

In Cambodia, the NMP’s DTWG is responsible for initiating the process of reviewing anti-malarial policy in response to new evidence regarding drug resistance, changes in WHO guidance and/or difficulty procuring currently recommended treatment. The DTWG’s membership includes representatives from various NMP departments, the WHO Cambodia office, implementing and research partners and funders. A larger group of technical experts provides input to inform proposed policy changes during the annual Antimalaria Drug Policy Meeting. The Drug Policy Meeting is larger than the DTWG, attended by the full DTWG as well as additional representatives from NMP and the Ministry of Health, WHO representatives from global, regional and country offices, funders, research and implementing partners. The Minister of Health is responsible for approving proposed changes to the malaria policy in Cambodia. The Ministry of Finance is not consulted, but donor organizations, including Global Fund and the President’s Malaria Initiative (PMI), are described as having significant influence over policy change decisions in Cambodia.

#### *Process: pathway & timeline for policy change*

**Fig. 1:**
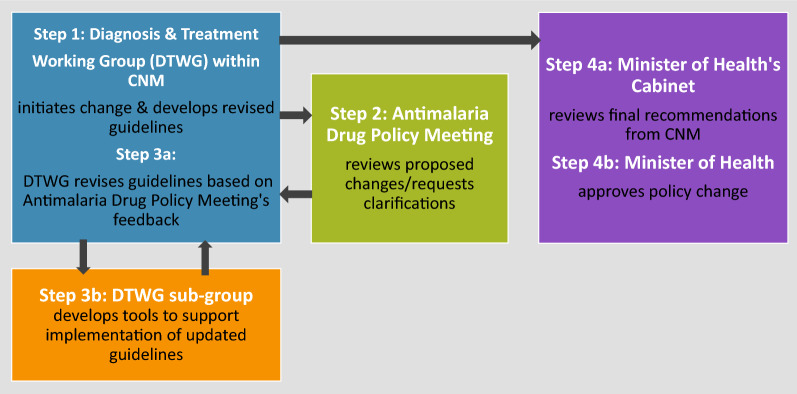
Key steps in the policy change process in Cambodia

Cambodia’s policy review process begins with multiple DTWG and sub-DTWG meetings during which available national and global evidence is reviewed and used to inform efforts to draft suggested revisions to the guidelines. While the DTWG ToR refers to monthly meetings, between October 2019 and March 2020, the DTWG met approximately 3 times—roughly half as frequently as mentioned in the ToR. During the same period, there were several smaller, sub-DTWG meetings to discuss detailed planning for a vivax malaria pilot project launched in late 2019. Once consensus within the DTWG is reached regarding the required policy changes, revised guidelines are drafted and presented for validation at an “Antimalarial Drug Policy Meeting”. This meeting is held annually in May or during non-routine times as requested. After the Antimalarial Drug Policy Meeting a smaller, sub-working group is responsible for finalizing guidelines consistent with feedback collected through this meeting. NMP submits guidelines for review by the Minister’s Cabinet before receiving official endorsement by the Minister (Fig. 1).

In cases where revised guidelines require a drug that is not yet registered in Cambodia, then the NMP will support registration by writing to the Minister of Health requesting and explaining the importance of fast-tracked regulatory approval, providing that the drug in question is already prequalified by the WHO. If the Ministry agrees with the justification, they will ask the Department of Drugs and Food (DDF) to accelerate review of the dossier submitted by the manufacturer. According to the NMP respondent this scenario rarely happens since the WHO is integrally involved in Cambodia’s policy change process and generally guides the process to focus on previously approved and registered drugs.

Based on respondent’s experience, 6–12 months is typically required to change anti-malarial policy. The latter is reviewed more regularly compared to other diseases because of the speed with which drug resistance has evolved compared to other diseases like tuberculosis. Planning, preparing, and budgeting to execute policy changes can take one year, representing half to one-third of the total policy change timeline. Respondents were not aware of whether or how the malaria policy process differs from the process for other public health priorities such as tuberculosis or HIV/AIDS. Ministries or partners outside of the health sector are not included in the Antimalarial Drug Policy Meeting or other parts of the policy change process.

### Ethiopia

#### *Context*

The socio-economic context and the current status of anti-malarial malaria policies are summarized in Table 2. In terms of funding for malaria control activities the Global Fund plays a key role with some smaller funding being provided through USAID/PMI and other sources. The process used to review and revise diagnosis treatment guidelines in Ethiopia is not documented by the Ministry of Health. Documented Terms of Reference are not available for the Technical Advisory Committee (TAC) or the Case Management Technical Working Group (CMTWG), both of which play significant roles in the policy-making process.

#### *Actors: responsibility for initiating, reviewing and approving policy change*

Ethiopia’s TAC—under the NMP’s guidance—initiates policy changes in Ethiopia, based on changes to WHO guidance or new data received from Regional Health Bureaus or research agencies. The TAC is co-chaired by the NMP Coordinator and the USAID President’s Malaria Initiative (PMI) with a representative from the Malaria Consortium currently serving as Secretariat. This advisory forum includes approximately 15–20 participants representing 10–15 implementing organizations and research agencies as well as NMP representatives. The TAC meets approximately once every 1–2 months, although it can meet more regularly if requested by the NMP. The Case Management Technical Working Group (CMTWG) is a smaller group, linked to the NMP, responsible for reviewing available evidence in detail and developing updated guidelines consistent with local evidence and/or WHO guidance. At a given CMTWG meeting, approximately 6–8 participants are from the NMP, the Ethiopia Public Health Institute, the WHO, financing partners and specific international research institute projects. The Ministry of Finance is not consulted, but external financing partners are described as having significant influence over policy change decisions in Ethiopia.

#### *Process: pathway and timeline for policy change*

**Fig. 2:**
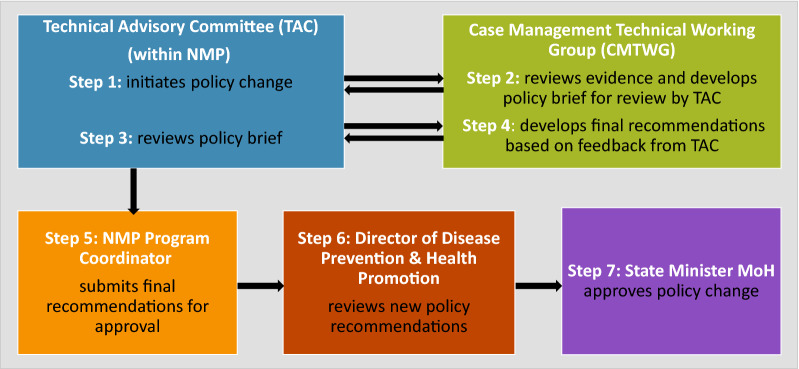
Key steps in the policy change process in Ethiopia

Malaria guidelines are generally reviewed every 3–5 years in Ethiopia, typically in response to updated guidance from the WHO or new data relevant to malaria guidelines—usually generated by Regional Health Bureaus or research agency partners. The CMTWG develops a policy brief—summarizing the evidence base and proposed updated guidelines—and submits this to the TAC for review. The TAC’s review may lead to questions and requests for clarification or revision to the policy brief. Once the TAC is satisfied, the TAC notifies the NMP Coordinator to submit for approval by the State Minister. The approval process involves the NMP submitting a written request for approval to the Director of Disease Prevention and Health Promotion for review before the Director passes the guidelines to the State Minister for approval (Fig. 2). The respondents stated that the MoH would rarely decide not to approve guidelines put forward by the NMP since the technical nature of this guidance falls within the NMP’s mandate. No other Ministries or Government agencies are involved in the malaria policy-making process.

In cases where guidelines require a drug that is not yet registered in Ethiopia, the State Minister can approve guidelines referencing a drug that is not yet approved by the Ethiopian regulatory body. However, before a drug can be imported, approval is required from the Ethiopian Food and Drug Administration (FDA). Where a new drug is included in approved malaria guidelines, the process of registering this drug with the Ethiopian FDA will take into consideration previous technical guidance and approval from the NMP and State Minister respectively.

Respondents estimated that the time required to change policy was between 6 months to 2 years. If the CMTWG determines that additional research is required to inform policy change, the process may take up to 2 years. If the policy changes are consistent with recent WHO guidance and additional local research is not required, the process requires less time. Stakeholders interviewed were not aware of whether or how the anti-malarial policy process differs from the policy process for other public health priorities such as tuberculosis or HIV/AIDS.

### Indonesia

#### *Context*

Country specific context regarding current treatment guidelines and the socio-economic context are summarized in Table 2. Domestic funding for malaria control activities has been a key mechanism in Indonesia for more than 10 years and is currently contributing approximately 40% to the overall budget [18]. The process used to review, and revise diagnosis treatment guidelines is not documented. The main actor to drive the process is the National Malaria Expert committee for diagnosis and management, and the national malaria programme. The expert committee has ToRs that are documented in the Ministry of Health’s decree for the Diagnosis and Treatment of Malaria Working Group [48].

#### *Actors: responsibility for initiating, reviewing and approving policy change*

In Indonesia, policy process is initiated by the National Malaria Expert committee for diagnosis and management if the policy is related to diagnosis and treatment. The committee is invited by the national malaria programme to meet with its representatives to review available evidence. Members of the expert committee are appointed by the malaria programme and include researchers, clinicians and provincial representatives (a total of 25 to 30 people). The committee meets at least twice-yearly to review evidence. Other expert committees with specialist expertise to consider evidence and relevant materials may also be consulted on the policy process as well as other relevant stakeholders depending on the type of policy change. If urgent, additional meetings can be arranged by the programme or requested by the expert committee. The committee produces yearly updates regarding efficacy and other relevant evidence collaboratively with related programmes and institutions such as the National Health Research Institute and Development, the Eijkman Institute, the Indonesian Food and Drug Authority (BPOM), national medical associations or related professional organizations and the Maternal and Neonatal Health Programme. Small group discussions are also undertaken before these recommendations are adopted into policy. The Director of vector borne and zoonotic disease prevention’s Directorate General is ultimately responsible for the approval of a revised policy.

#### *Process: pathway and timeline for policy change*

Recommendations drafted by the National Malaria Expert committee for diagnosis and management are approved by the expert committee head and sent to the national malaria programme. Thereafter, the malaria programme sends a letter of recommendation to the Director of vector borne and zoonotic disease prevention’s Directorate General. Concurrently the malaria programme manager also provides a brief verbal overview of recommendations to the Director of vector borne and zoonotic disease prevention.

**Fig. 3:**
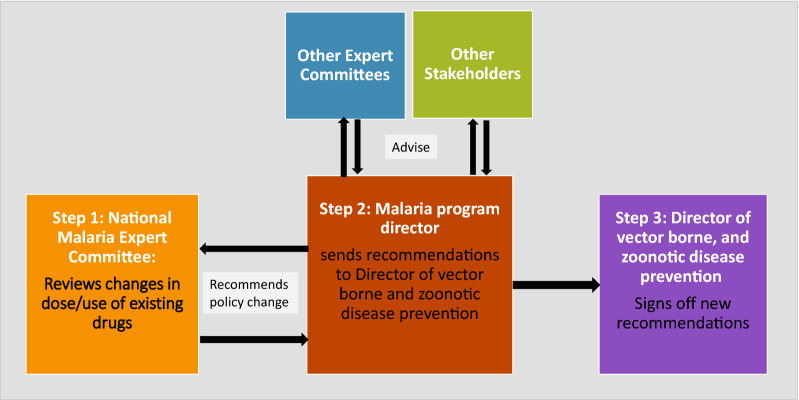
Key steps in the policy change process in Indonesia

The malaria programme and expert committee then co-draft revised treatment policy guidelines. The malaria program finalizes guidelines and sends these back to the committee for rechecking. Most of this communication is conducted via a WhatsApp group. Respondents stated that this allows for dynamic discussion and timely resolution. In urgent circumstances (such as guidelines for Malaria Service in COVID-19 Pandemic Situation which includes treatment guidelines for malaria patients with COVID19), these reviews happened within days. Drafted guidelines are then reviewed by the malaria programme, other relevant stakeholders and other expert committees (for e.g., the committee for operational research on malaria). Final revised guidelines are then sent to the Director of Vector borne and Zoonotic Disease Prevention and Control with a recommendation summary for sign off (Fig. 3).

In the case of introduction of drugs that have not been used previously in country, stakeholder consultation includes the BPOM (including sub-committees such as drug product and evaluation working group) and the Directorate of Pharmacy and Medical Devices (under the MoH) and sign off happens at ministerial level. The Ministry of Finance is not directly involved in the policy process.

Based on experience to date, the maximum length for a policy change in Indonesia is 1 year. In most cases it requires only 6 months. The two last significant changes were in 2004 from chloroquine (CQ) to amodiaquine (AQ) and in 2006 when Dihydroartemisinin-Piperaquine (DHAP) was introduced [49]. The second change occurred prior to WHO recommendation of DHAP but this process was tabled confidentially, therefore its documentation into policy cannot be directly traced. Respondents were unsure about the policy process for other disease programmes.

### Pakistan

#### *Context*

Country specific context regarding current treatment guidelines and the socio-economic context are summarized in Table 2. Nearly 50% of the funding for anti-malaria activities are covered through domestic resources [18]. Guidance for policy change processes or documentation of previous policy changes was not available. The Technical Working Group (TWG) within the National Malaria Programme (Directorate of Malaria and other Vector Borne Diseases (DoMC)) meeting minutes document some aspects of the policy change process, but the steps and responsibilities in the process are not outlined in any official document.

#### *Actors: responsibility for initiating, reviewing and approving policy change*

The TWG is the primary body responsible for the malaria policy change process in Pakistan. The TWG meets at least twice yearly to review evidence relevant to possible policy changes and discuss other programmatic issues, although more regular meetings can be organized as needed. Agendas are developed with input from members including representatives from the case management and surveillance subdivisions within NMP, WHO, a key financing partner and the Indus Health Network, which operates public health outreach programmes linked to private health facilities in Pakistan. Chaired by the Director of the NMP, the TWG’s mandate is to review available evidence and translate relevant research findings into recommended policy changes.

#### *Process: pathway and timeline for policy change*

The policy change process in Pakistan requires only few steps. The NMP initiates, reviews and approves anti-malarial guidelines. The TWG within the NMP is responsible for reviewing the available evidence and WHO guidance to inform updated guidelines. TWG submits revised guidelines to the Director of the NMP for approval. No other Ministry is involved in the malaria treatment policy process. WHO guidance is described as highly influential in Pakistan, and in most cases the guidelines are updated following updates to WHO guidance or local therapeutic efficacy study findings.

In cases where revised guidelines require a drug that is not registered in Pakistan, the NMP can submit a written justification to the Drug Regulatory Administration in Pakistan (DRAP) to inform their review of the dossier for a new drug. The NMP may be involved in multiple meetings with DRAP to discuss questions related to the submission of a written justification for local approval of a new malaria drug. In some cases, the NMP will also advocate to the Director General or the Secretary of Health in the Ministry of Health, requesting a letter from the MoH to the DRAP in support of approval of a new malaria medicine.

Based on experience to date, approximately six months is required to change malaria treatment policy in Pakistan. More time is required if the proposed changes are not consistent with recent WHO guidance updates. Respondents explain that to date, most policy changes have followed the release of updated WHO guidance. They were not aware of whether or how the malaria policy change process differs from the policy change process for other health areas.

### Papua New Guinea

#### *Context*

Country specific context regarding current treatment guidelines and the socio-economic context are summarized in Table 2. Almost all funding for the malaria programme in Papua New Guinea (PNG) comes from the Global Fund [18]. There was no guidance for the diagnosis and treatment guideline change process in PNG, although the 2021–2025 national malaria strategy document (under development at the time this paper was drafted) references the Technical Working Group’s (TWG) role and some aspects of the policy change process.

#### *Actors: responsibility for initiating, reviewing and approving policy change*

The policy change process in Papua New Guinea is coordinated by the Malaria Programme within the National Department of Health (NdoH) and involves consultation with several stakeholders including paediatricians and other “specialist” medical societies, research agencies, the WHO and others. Within the NMP, the Technical Working Group is responsible for initiating the policy change process in PNG. The push for initiating a policy review typically comes from questions raised through this group regarding drug efficacy and/or new WHO recommendations. The TWG was created in 2010 with a mandate to meet every 2 weeks and is chaired by the NMP Director. There are approximately 15–20 members of the TWG including NMP technical experts, WHO, the Global Fund Prime Recipient and others directly involved in managing malaria programmes in PNG. Other partners are invited as needed, to present data relevant to and/or discuss specific issues. Once the TWG agrees that guidelines need to be revised, they organize a larger group of technical stakeholders to review policy revisions drafted by the TWG. Stakeholders involved in the policy review process include the TWG members, as well as key specialist medical groups including paediatricians, gynaecologists, and physicians, as well as the WHO, research agencies and implementing partners. Other Government agencies/bodies are engaged depending on the nature of the proposed policy changes. For example, the Central Public Health Laboratory is responsible for validating operations research findings and policy recommendations related to G6PD testing. Participation in the policy review process is not restricted in PNG, global health partners, research agencies and implementing partners are welcome to participate in the technical consultations preceding policy change.

Key influencers in PNG’s policy change process include heads of the paediatrician and physician specialist groups, the Pharmaceutical Services Department and WHO. The WHO serves in a recognized, technical advisory capacity throughout the process including during both the initiation and review sub-processes. Consultations with influential paediatricians often take the most time as they are seen as risk averse when reviewing potential policy change. This group typically has numerous concerns and questions regarding proposed changes to the D&T guidelines, particularly malaria treatment regimens for children. Recently, proposed changes to treatment guidelines did not initially specify dosage for children under five, and the NMP was asked to add treatment guidelines for this age group which involved extensive discussions with paediatrician groups, given the complexities of identifying safe and feasible treatment for children requiring partial tablet dosage.

#### *Process: pathway and timeline for policy change*

**Fig. 4:**
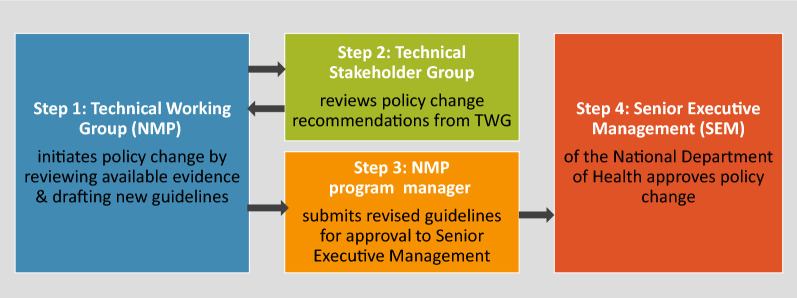
Key steps in the policy change process in Papua New Guinea

TWG decisions and deliberations—including initiating guideline review—are made consultatively and documented through meeting minutes. Review of guidelines in PNG requires consultation with 30–40 experts culminating in a five-day workshop to discuss the proposed new guidelines and the supporting evidence base. Once input from the larger group of technical stakeholders has been incorporated, the NMP Manager submits revised guidelines for approval to the Senior Executive Management (SEM) within the National Department of Health. The SEM organizes and documents reviewer feedback from various divisions within the Department of Health before formally approving new guidelines. In most cases, SEM queries are requests for clarification purposes as technical recommendations from the NMP are rarely rejected. The TWG ToR—in development at the time this research was conducted—will clarify the TWG’s ability to make decisions based on technical grounds before requesting SEM endorsement. Final policy approval by the SEM is documented through minutes of endorsement from various reviewer divisions including public health, curative health, and other reviewer divisions (Fig. 4). The hierarchy and internal dynamics within the National Department of Health also influence policy change process in PNG. At present, the PNG policy change process is viewed as a collective decision made by the larger group of medical researchers and experts consulted under the NMP’s leadership. Proposed changes need to be supported by evidence which typically involves presentation of feasibility study findings to the larger expert group for review. Feasibility studies are not typically designed according to evidence that expert reviewers think is needed.

If the proposed policy changes require a new medication that is not yet registered in PNG, the new drug registration process also needs to be navigated. Only medications included in PNG’s catalogue listing of all approved medication are listed with the medical stores system. The Medicine Quality Sub-Committee within the National Department of Health’s Pharmaceutical Services Division is responsible for reviewing proposed additions to the approved medicines list for PNG. The Pharmaceutical Services Division also influences the approval of new diagnostic tools.

The time required for the full policy change process can be two to three years. The process of developing current guidelines started in 2008 and ended in 2011. If paediatricians and other key stakeholders are not consulted early in the process, and/or if the available evidence is viewed as insufficient to support the recommended policy change, the process can be extended. Policy changes regarding new or modified diagnostics can be actioned based on NMP recommendations alone, and do not require the full review process used for changes to treatment regimen. The Ministry of Finance is not consulted during the review process. Financial stakeholders within and beyond the Ministry of Health are consulted after the guidelines are revised, during budget and procurement-related discussions. Respondents were not sure whether the malaria policy change process differs from the process for other health areas in PNG.

### Sri Lanka

#### *Context*

Country specific context regarding current treatment guidelines and the socio-economic context are summarized in Table 2. There was no guidance available for the policy change process. Additionally, whereas policy change processes have not previously been documented, the country’s national strategic plan (NSP) for malaria refers to the Technical Support Group (TSG)’s mandate, membership and 2017 terms of reference [47].

#### *Actors: responsibility for initiating, reviewing and approving policy change*

The TSG plays a significant role in Sri Lanka’s policy-making process and meets every 3 months under the chairmanship of the Director General of Health Services. TSG members are appointed by the Director General and typically include technical experts and representatives from the National Malaria Programme (the Anti-Malaria Campaign (AMC)) and other divisions within the MoH and the Health Promotion Bureau, a representative from the WHO as well as Regional Malaria Officers, senior parasitologists, entomologists, clinicians and pharmacologists, several of whom are also professors from leading universities. The policy change process involves consultation with four technical stakeholder forums within the AMC: the Case Review Committee (CRC), the Regional Malaria Officers (RMOs), the AMC Technical Officers and the Drug and Therapeutics Committee (DTC.)

**Fig. 5:**
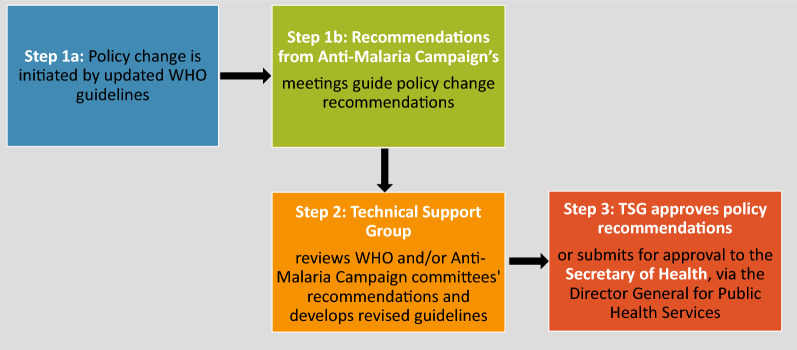
Key steps in the policy change process in Sri Lanka

The TSG is responsible for reviewing the evidence base, including updated WHO guidance, to inform proposed changes to malaria treatment policy (Fig. 5). In some cases, the TSG Chair can approve guidelines and in others the Secretary of Health approves through the Secretary of Public Health Services. The TSG determines, through consultation within the TSG, whether guidelines require Director General-level review. Generally, changes based on straightforward technical updates—such as a revision aligned with updated WHO guidance—can be approved by the TSG Chair. But in cases where implementation of the policy change will affect multiple Ministries, a higher-level approval is recommended.

In cases where new drugs are required by the updated guidelines, the Drugs and Therapeutic Committee (DTC) must approve a new drug before it can be included in the guidelines. In these cases, the TSG will advocate—both formally (in writing) and informally (through TSG members who are also DTC members)—to the Director of the Drugs Regulatory Authority to advocate for approval. Typically, 3–4 months are needed for the DTC to review and approve a dossier submitted by a pharmaceutical manufacturer.

#### *Process: pathway and timeline for policy change*

The process used to review and revise malaria guidelines in Sri Lanka is relatively straightforward and requires less time compared to the process in other reviewed countries. The impetus for changing malaria policy in Sri Lanka stems from needs identified by Sri Lanka’s Prevention of Reintroduction of Malaria Programme and WHO guidance. All recent revisions to Sri Lanka’s policy were guided by updated WHO guidance. Each of the technical forums within the AMC—involved in the malaria policy change process—meet regularly, and meeting discussions through these forums feed into the policy change process managed by the TSG. The CRC is chaired by the Director of the AMC and includes senior professors of parasitology, pharmacology, clinical medicine, and other AMC technical staff. The CRC meets monthly to discuss case finding data, often together with the RMOs. Based on the CRC’s detailed review of each case, this body recommends corrective measures needed to the TSG as needed. The RMOs meet every 2 months to discuss district-level data and priorities. The Technical Staff within the AMC meet monthly to review surveillance, monitoring and evaluation data. Any issues identified through this meeting are submitted to the TSG to inform policy decisions. The DRC meets monthly to discuss procurement of malaria commodities with input from the Medical Supplies Division within the MoH as well as the State Pharmaceutical Corporation (SPC). The DRC also shares information with and makes related recommendations for the TSG’s review and guidance. One way the malaria policy pathway differs from other disease areas in Sri Lanka relates to the TSG’s ability to incorporate input from professional colleges most relevant to proposed policy changes.

Sri Lanka requires 3 months, on average, to change anti-malarial policy. While consultation with the Ministry of Finance and/or other Ministries is not a standard part of the policy change process, if necessary, MoH can request inputs from others. Several factors explain the brevity of Sri Lanka’s policy change process. The consolidation of policy change initiation, review and approval within the TSG for most cases translates into a relatively condensed set of steps in the policy change process. Second, the emphasis on WHO guidance to inform policy change, limits the requirement for additional, local research to be conducted to inform policies. Third, the inclusion of representatives from the Medical Association and the College of Physicians in the TSG reduces the risk of delays, as questions from these key influencer groups are raised relatively early in the process.

### Vietnam

#### *Context*

Country specific context regarding current treatment guidelines and the socio-economic context are summarized in Table 2. Approximately three quarters of the malaria funding comes from external sources with the Global Fund being the most important funding source [18]. Vietnam’s policy change process was in the process of being documented in late 2020 when data was collected for this paper.

#### *Actors: responsibility for initiating, reviewing and approving policy change*

The malaria policy-change process in Vietnam involves multiple Government agencies including, but not limited to the National Malaria Programme (National Institute of Malariology, Parasitology and Entomology (NIMPE)). Whereas the NMP is responsible for initiating policy change, multiple other divisions within the Ministry of Health are involved in reviewing the evidence base and proposed policy changes before approval. Malaria case management guidelines are reviewed at least every 2-years, with interim reviews possible in response to evidence of resistance. For example, the 2019 malaria policy review was initiated following evidence of delayed parasite clearance in two provinces. In cases where new drugs are required by proposed guidelines, the Drug Administration of Vietnam’s approval of new drugs is required. Draft guidelines are reviewed by an MoH Expert Committee including representatives from Vietnam Administration of Medical Services (VAMS), the General Department of Preventive Medicine, the MoH’s Training & Research Department, the Drug Administration of Vietnam, national hospitals, universities, the WHO, the NMP and regional Malaria Programmes (MPs). The Expert Committee’s composition and meetings are guided by VAMS—participation varies from one review to another. The WHO is highly influential in Vietnam’s policy change process. The Ministry of Finance is not consulted during the policy change process in Vietnam, although recent policy changes were reportedly influenced by budget considerations. Specifically, the costs associated with roll-out of comprehensive G6PD testing influenced the decision to recommend but not require G6PD testing in the pending 2020 guidelines.

#### *Process: pathway and timeline for policy change*

The NMP initiates policy change in response to evidence identified through discussions during their monthly meetings and scientific seminars through which NMP leaders and technical experts receive updates on all research including therapeutic efficacy studies and province-specific monitoring data. In response to evidence of resistance or a change in WHO recommendations, NMP’s Institutional Review Board (IRB) is tasked with reviewing the available evidence and working with NMP technical experts to draft updated guidelines. The NMP’s IRB organizes a review by the Scientific Committee involving 19 experts from NMP, research agencies, provincial governments and WHO. Feedback from Scientific Committee is incorporated into revised guidelines through a series of meetings and other consultations. In some cases, questions by the Scientific Committee members are resolved quickly, but in other cases it requires additional meetings to address all questions. If the evidence-base in support of revised guidelines is not clear the Scientific Committee may ask for additional research to be planned and conducted which can extend the timeline significantly.

**Fig. 6:**
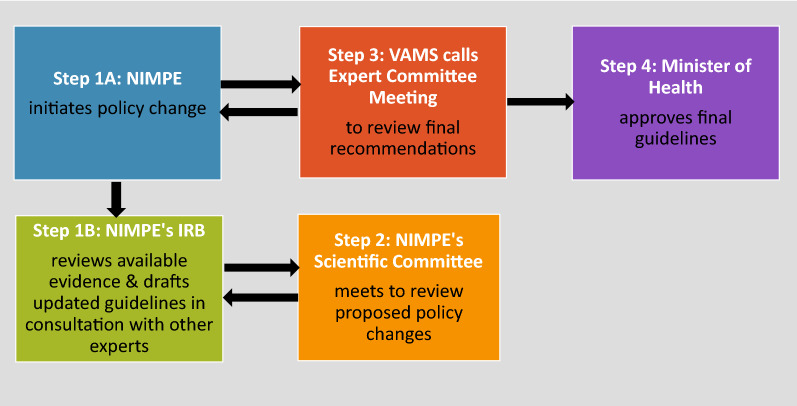
Key steps in the policy change process in Vietnam

Once NMP’s Scientific Committee has endorsed the proposed guidelines, the NMP Director submits proposed guidelines for review by an Expert Committee organized by VAMS. NMP is asked to respond to questions from Expert Committee reviewers before VAMS compiles and submits final guidelines for approval by the Minister of Health. Guidelines are submitted by VAMS for approval by the Minister of Health (Fig. 6). Local clinical trials can be initiated prior to registration. WHO approval of proposed new drugs is highly influential over DAV registration and policy change decisions in Vietnam.

Time required for the full policy change process in Vietnam can be several years, depending on whether additional research is required to validate efficacy or safety of proposed changes. It was unclear from the interviews conducted whether the malaria policy change process differs from the process for other health areas in Vietnam.

### Cross country comparison: commonalities and differences

#### *Process documentation and clarity*

The seven countries included in the study represent distinct contexts in terms of socio-economic status of their population, population size and funding sources for malaria programmes. Only two countries, Cambodia and Indonesia were able to provide ToR documentation for the Technical Working Group responsible for reviewing and/or recommending policy changes. None of the seven countries were able to provide a document summarizing the full policy change process or describe if their respective MoH had a standard process.

#### *Local evidence and policy change*

The importance of local evidence and global endorsement of new treatment and diagnostics by the WHO varies. In some countries such as Sri Lanka, policy change predominantly follows WHO guidance. In others like Pakistan, local evidence of the feasibility and safety of WHO guidance is also required before policy changes can be made in line with global recommendations. In Indonesia, the expert committee focuses on in-country local evidence. WHO recommendations are also considered but need to be supplemented by in-country evidence to provide a sound evidence base for policy change. If the body of in-country evidence is robust, policy change can be initiated before WHO recommendations are made.

#### *Decision initiators and influences*

In all countries, the NMP plays a critical role in initiating and informing policy change, however, the extent to which other Government departments, agencies and ministries are involved in reviewing or approving recommended guidelines differed. For example, in Pakistan, the NMP has primary responsibility for initiating, reviewing and approving malaria policy changes. By contrast, in Vietnam, the NMP initiates policy change, but review and approval of policy changes take place outside of the NMP by other divisions within the MoH including the Department of Preventive Medicine and the Vietnam Administration of Medical Services.

Research agencies and WHO are key influencers in this process across all seven countries. External donors are highly influential, particularly in countries with national malaria programmes heavily dependent on external funding such as PNG and Ethiopia. The policy process itself is relatively fluid in all countries, depending on the scope of the policy change recommended and related considerations.

At present, none of the countries consulted with the Ministry of Finance during the policy change process, which means that budgetary implications of policy change may only be partially considered. In some countries like Ethiopia, drug policy can include medicines not yet locally approved by the regulator. However, in most cases local registration is a precondition for any medication included in national guidelines. The Pakistan policy pathway represents a case where the NMP is more engaged in advocating for approval of new anti-malarial drugs, with local regulatory bodies and influential MoH leaders. Information on policy pathways for drugs that require registration (e.g., TQ) was limited and knowledge about those pathways was often outside the expertise of the respondents. As such, policy pathways described in this paper focus on guideline changes regarding usage of currently licensed drugs (e.g., PQ). Reported policy change timelines varied considerably between countries from 3 months to up to 3 years reflecting a high degree of variation.

## Discussion

Policy pathway processes in seven vivax malaria endemic countries with different health systems and socioeconomic and political contexts were mapped, using the framework of context, actors and process to structure and interpret the country specific results. Results suggest revision of anti-malarial policy for *P. vivax* could be hampered by under documented, complex and time-consuming policy-making processes. Except for Vietnam, NMP representatives and their stakeholders were largely unaware of the MoH policy-making processes beyond malaria, suggesting that in this space, decision-making seems tangential to the MoH as a whole. However, the triggers for policy change appeared to be uniform across all countries—occurring primarily in response to updated WHO guidelines or new data becoming available. However, the decision-making space in each of the studied countries lacked overall clarity and specificity even when documentation was available (for instance ToRs for decision-making bodies). The length of the policy change process is highly variable both within and between countries, likely dependent on the impetus for policy change, whether new products are being incorporated and whether proposed changes have already been globally approved by WHO. Requirements for nationally generated evidence is also variable.

The impact of the political and financing contexts that influence each country’s malaria policy-making processes are important factors to consider when appraising policy-making processes. While epidemiological context is most likely given consideration in each country, the influence of political and economic context is harder to gauge and less often acknowledged as an influential factor on national policy processes. However, in Africa the significance of these two factors has been highlighted to improve health policy processes drawing attention to the influence political will, MoH and National Malaria Programmes’ (NMP) leadership, and cost implications have on changing national policy processes [10, 11, 50]. Similar influences may also shape anti-malarial policy-making landscape in countries discussed here. The WHO, external funders and research agencies for example, play influential roles in malaria policy-making processes in most of these countries. The substantial influence of global recommendations in some countries may be at least partially caused by a reliance on external funding that influences decisions by NMPs [51–53]. This might be compounded by time/bandwidth constraints for NMPs coordinating a wide range of implementation activities, partners and funders while simultaneously meeting internal (MoH) reporting needs.

In most countries policy processes are not transparent, with a lack of guidance to the steps of the process and the documentation of previous policy change processes. This opaqueness has been described by Walt and Gilson and more recently others as the “black box” of policy-making processes [5–7]. Dodd et al. specifically highlight the complexity and lack of clarity of national health policy processes in Bangladesh which may be comparable to the situation in the countries included in this research, given similar contextual factors [51]. The potential impact of opaqueness of policy processes on national health outcomes is unknown. It is unclear from this research, whether decisions about malaria and other health policies are the best decisions that could be made at that point in time, with the evidence that is available. Increasing the transparency of policy processes to be able to understand the influences on those decisions could ensure more accountability, more timely appraisals of options and ultimately, decision-making for better health outcomes.

A lack of clarity on the respective roles of key decision-makers in the policy change process was identified. Yet, there are numerous actors including financing partners, research groups, implementing partners, non-governmental organizations and technical agencies that can potentially influence decision-making at different points in the policy cycle. Previous research in Cambodia and Pakistan highlights that external financing partners wield significant influence in national policy-making processes because they were perceived to have greater technical expertise compared to national policy actors and by directly controlling available finances [52]. This study also confirms that in most of these countries WHO and in some instances, funders are key actors in the policy-making process. Hence while NMPs are the key body with influence over decisions, they are not always the primary decision-makers driving change. Rather they are guided and, to an extent, take on the role of implementers more than key decision-makers which may or may not be in the best interests of a population’s health.

Documented, explicit ToRs for the technical advisory and policy review or approval committees could potentially enable NMPs and the MoH to better weigh external influence of technical agencies, research agencies and external donors in the decision-making space. ToRs could more explicitly describe requirements for diversity in composition of decision-makers in terms of, for example, type of expertise provided, and declaration of any conflicts of interest. Relatedly, and in recognition of poor representation of women in global health organizations (approximately 30% in leadership positions), and specifically women from Low to Middle Income Countries—LMIC (approximately 5%) in leadership and decision-making roles, clearer ToRs could also include specific benchmarks to address these and other social inequities [53]. In the case of vivax malaria, a lack of diversity in the overall decision-making process may also result in less focus on addressing the needs of specific populations such as G6PD heterozygous females [54]. More importantly it might lead to narrow decision-making at the cost of already marginalized groups.

Variations in the importance of nationally generated evidence from country-to-country also influence policy change. Some countries, like Sri Lanka, prefer to wait for new WHO recommendations to change policy while others, such as Indonesia, may change guidelines based on local evidence alone if it is considered robust. A question remains as to how countries define robust and how much and what evidence is needed before a policy change is implemented nationally. Policies that are flexible and adaptable to different contexts and situations seem the best approach as recent rapid response approaches to COVID 19 and other policy adaption literature suggest [55–58]. Experience from anti-malarial treatment policy change from CQ/SP to AL in Uganda showed that “contextualized evidence” was needed to effectively change policy [59]. In this case, it was by ensuring that presented evidence supported economically feasible policy-making and responding to community feedback on new drug regimen piloting before finalizing new malaria treatment policy guidelines and changeover to the new treatment.

Policy change takes time and, in some of the countries in this study, an inordinate amount of time. What is considered an *appropriate length of time* to propose, review and approve a change in policy needs urgent reflection if countries are to meet their elimination targets. The longer a policy takes to change, the greater the negative impact on population health [34]. Policy change pathways of other disease programmes provide examples of streamlined processes and cross health sector collaboration which could be employed by NMPs to enable faster malaria policy change. For example, rapid policy change within a year of India’s tuberculosis (TB) and diabetes screening guidelines in 2012 to a bi-directional screening process where multiple national organizations and international stakeholders including WHO and the World Diabetes Foundation were integral in cross health sector collaboration, screening guidelines and pilot programme appraisal prior to policy change [60].

Rapid introduction of policies on COVID-19 guidelines for diagnostic testing and screening, patient classification, priority setting for hospital bed allocation and preventive measures in South Asia and the Middle East highlight that accelerated policy change is possible within a span of several months to a year when political will supports streamlined policy-making processes [61–63]. Yet, *how* that political will is developed *from within the region* and maintained as malaria numbers are dwarfed by other diseases and conditions, such as COVID or non-communicable diseases, is a major challenge facing the Asia Pacific region as countries edge closer to elimination. COVID-19 is an unprecedented new global health challenge with a different epidemiological profile and political and economic context to malaria. However, lessons could be learned in areas where novel policy innovations were used for COVID, including faster, more transparent and adaptive decision-making [57, 64, 65]. However rapid policy change can result in significant drawbacks, such as the conflicting policy messaging on the usefulness of wearing face masks [66–68]. This begs the question of how to introduce new policy with appropriate speed, which is communicable, and potentially reversible i.e., adaptable based on emerging evidence [37, 69, 70].

Generally, across the included countries there was limited connection between NMPs and MOHs in terms of policy-making. This work highlights that anti-malarial policy-making processes are sometimes semi-siloed from MoH processes. This structure is par for the course in the malaria world where programmes with a high ratio of external funding, are highly vertical facilitating ease of coordination with financing partners often bypassing national processes but whether it should be so remains a question [71–74].

Results suggest that consideration of cost-effectiveness or budget impact is not part of the process when deciding to change treatment policies in the countries included in this analysis. One reason for this maybe reliance on external donors to fund malaria interventions and therefore limited need to consider domestic financial implications. Furthermore, partners assisting in the development of funding proposals may provide an overview of the cost-effectiveness of new tools as they relate to financing partner interests (e.g., Global Fund’s Value for Money framework) but unrelated to the potential impact on the NMP or MOH budgets. Irrespective, the lack of internalization of the costs of a potential new policy by NMPs and other stakeholders involved in the policy pathway is concerning and may indicate limited recognition of the need or possibilities to increase domestic budgets for malaria, potentially explaining, at least in small part, the stagnation in domestic funding globally for malaria in recent years [75].

There are several limitations to this work. Firstly, results are based on input from a select number of key informants with different roles within NMPs and some additional key stakeholders. This could have led to a lack of awareness of all available documentation regarding process and actors. However, all interviewees were senior personnel with in-depth knowledge and experience in their field. In-depth interviews were conducted to further cross-check and validate the initial policy process maps. However, more in-depth interviews with additional stakeholders including non-NMP respondents involved in the policy pathway to determine how the policy process *actually functions* versus how it *functions in principle* was outside the scope of this study. Secondly, documentation relevant to policy change processes and actors was requested from interviewees. However, no guidance as to the steps in the policy change process, or benchmarks as they relate to the development of ‘good’ policy could be provided. As a consequence of this and an overall lack of documented national policy change processes, ToRs for decision-making committees from only three countries—Cambodia, Indonesia and Sri Lanka—were available to cross-check against verbal information provided by interviewees. Thirdly, interviews with representatives from government offices or agencies outside of NMPs who are involved in the policy process were not within the scope of this study. Face-to-face consultation with these additional stakeholders and further document review could facilitate a more in-depth assessment of how policy change processes are actually implemented. Some suggestions for further in-depth research to address those limitations are listed in Table 3.

**Table 3:** Remaining questions

How do actual policy-change experiences and timelines compare to pathways identified?
Are there influencing bodies or partners not represented in formal pathways?
In cases where NMPs are involved in advocating for streamlined regulatory review of new malaria medicines or diagnostics, what are the specific approaches that have proven effective?
How do the pathways for policy and regulatory approvals intersect in different country contexts?
How long does each step of the policy-making process require on average?
Do the described decision-making pathways lead to good policy?
How do malaria policy pathways compare to pathways for other health programmes in the same countries? Are there opportunities to align malaria with national policy pathways for greater alignment with local and global good practices?
What is the gender and social inclusion breakdown of decision-making bodies involved in the policy pathway in each country?

## Conclusions

This study’s findings highlight that policy-making for vivax malaria and likely for other anti-malarial guidelines is characterized by under documentation and complex and often time-consuming processes. In the medium to longer term, better integration of policy-making processes for malaria into the overall national health policy-making processes (assuming those exist) would potentially strengthen overall health governance and would address the limited connection between the NMP and the MOH in the policy-making process [76]. Thereby ensuring any guidance provided to NMPs to facilitate their decision and policy-making processes is institutionalized within the MoH and aligns with its overarching guidance on policy-making and national health strategies [77, 78].

In the immediate term, timeliness is pivotal for countries attempting to meet their malaria elimination deadlines. Previous policy changes have not been well documented resulting in limited evaluation of the policy process and limited institutional memory. Little to no documented guidance was available for national programmes to facilitate their policy-making. The undocumented nature of these policy processes has potential to undermine policy analysis, decision-making and thus, timely implementation of optimal malaria control activities. A key conclusion from this work, that may help to address this is ensuring that documented guidance including best practice approaches is developed by countries to inform their policy processes.

## Data Availability

The datasets used and/or analysed during the current study are available from the corresponding author on reasonable request.

## Abbreviations

R&D: Research & Diagnostic
WHO: World Health Organization
PQ: Primaquine
TQ: Tafenoquine
PoC: Point of Care
CQ: Chloroquine
APMEN VxWG: Asia Pacific Malaria Elimination Network’s Vivax Working Group
IDI: In-depth interview
PNG: Papua New Guinea
NMP: National Malaria Programme
ToR: Terms of Reference
MoH: Ministry of Health
CNM: Cambodia’s National Center for Malaria
DTWG: Diagnosis and Treatment Working Group
TAC: Technical Advisory Committee
CMTWG: Case Management Technical Working Group
PMI: USAID President’s Malaria Initiative
TWG: Technical Working Group
Ethiopian FDA: Ethiopian Food and Drug Administration
DoMC: Directorate of Malaria and other Vector Borne Diseases
NdoH: National Department of Health
SEM: Senior Executive Management
TSG: Technical Support Group
AMC: Anti-Malaria Campaign
CRC: Case Review Committee
RMO: Regional Malaria Officers
DTC: Drug and Therapeutics Committee
SPC: State Pharmaceutical Corporation
VAMS: Vietnam Administration of Medical Services
NIMPE: National Institute of Malariology, Parasitology and Entomology
LMIC: Low to Middle Income Countries
